# From the archives: male–female communication, glue that keeps cells together, and a SUPERMAN for all flowering plants

**DOI:** 10.1093/plcell/koae019

**Published:** 2024-01-18

**Authors:** Marc Somssich

**Affiliations:** Reviewing Editor, The Plant Cell, American Society of Plant Biologists; Department of Plant-Microbe Interactions, Max Planck Institute for Plant Breeding Research, Cologne 50829, Germany

## April 2023: a role for the cytoskeleton in female–male communication

During sexual reproduction in plants, the male pollen delivers two sperm cells to the female ovule for fertilization. This is achieved via pollen tubes that are guided by attractant peptides to grow toward the ovule (Ogawa and Kessler 2023). Thus, female–male communication is required, which is in part facilitated by the synergid cell within the ovule. The synergid cell secretes the pollen tube attractants via a structure called the filiform apparatus (FA). As the FA is always established on the micropylar end of the synergid cells, both FA establishment and maintenance are dependent on cell polarity, which in turn is dependent on the cytoskeleton. Susaki et al. (2023) investigated the role of the cytoskeleton in forming and maintaining the FA in the ovule of *Arabidopsis thaliana*. Using genetic and chemical inhibitors of microtubule or actin network formation, the authors describe distinct roles for these two primary cytoskeleton components. Disruption of the microtubule network altered FA morphology, with reduced invaginations of the plasma membrane and cell walls, hinting at a role for the microtubules in cell shape mechanics. Disruption of actin, on the other hand, led to defects in directed cellular transport. As the establishment of cell polarity is dependent on the targeted transport of proteins, this also affected the formation and functionality of the FA, as well as the delivery and secretion of the pollen tube attractants. Thus, the actin and microtubule networks play distinct roles, but only actin is essential to facilitate male–female communication and fertilization.

## April 2019: how cells stick together

Moving from male–female to cell–cell connections, Šola et al. (2019) identify RUBY, a putative Gal oxidase (GalOx) involved in strengthening the pectin matrix of the cell wall. Plant cell walls are generally considered to be made of cellulose microfibrils that are cross-linked by xyloglucans to create a structural scaffold, which is embedded in a matrix of pectic polysaccharides and proteins. Among those features, the pectin matrix is particularly difficult to study, as it consists of a variety of different polysaccharides, which are further processed and cross-linked in various ways (Atmodjo et al. 2013). The *ruby* mutant, described in this 2019 paper, appeared to be impaired in such a cross-linking mechanism, as the mutant seeds showed cracks between cells, and particles could be observed in the otherwise homogenous mucilage secreted from the seeds (see Fig.). An analysis of the mucilage composition revealed that the mutant excreted certain pectin-derived cell wall polysaccharides that are normally retained in the wall of wild-type cells. The gene encoded by *RUBY* is a GalOx that acts specifically on the pectic rhamnogalacturonan I (RG-I), where it oxidizes galactan side-chains to cross-link carbohydrates and provide greater coherence to the pectin matrix. Thus, in the mutant, a weakening of the pectin matrix resulted in the excretion of sugars that would have otherwise been cross-linked to the cell wall and an impaired structural integrity of the middle lamella. As such, the ruby mutant is also a valuable genetic tool for further studies of middle lamella formation and maintenance.

**Figure.**
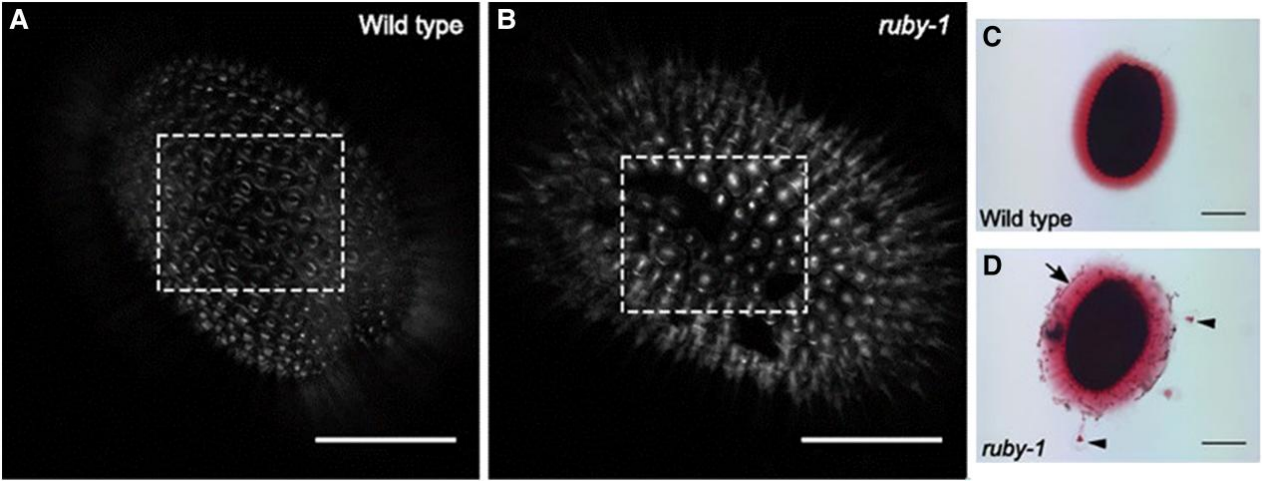
Phenotypes of *ruby-1* mutants. **A)** and **B)** RUBY is involved in cell-to-cell adhesion. Splits are visible between epidermal cells of *ruby-1* seeds (B) that do not appear in the wild type (A). **C)** and **D)***ruby-1* also exhibits mucilage phenotypes, including the appearance of particles in the excretions (black arrows in D). Scale bar, 200 *µ*m. Adapted from Šola et al. (2019).

## April 2004: a conserved role for SUPERMAN in flowering plants

Finally, before fertilization takes place in the ovule and seeds with polysaccharide-rich mucilage develop, there is usually a flower. The development of the ABC model to describe flower development in *A. thaliana* was one of the most important early findings that truly established *A. thaliana* as a plant model organism. Since then, it has been shown that the basics of the ABC model can be used to describe flower development in many flowering plants, among them *Petunia hyrbida* (Smyth 2023). SUPERMAN is a floral organ regulator that does not confer a specific identity to the organs of one of the four whorls but is required to establish the boundary between whorls three and four to separate stamens and carpels. In a 2004 study, Nakagawa et al. (2004) demonstrated that this is also true in *P. hybrida*, and they go on to describe some additional phenotypes of the Petunia *sup* mutant, particularly in placenta and ovule development. Follow-up studies on other flowering plants, including legumes and tomato, have shown that *SUP* and *SUP-like* genes are also involved in floral organ and fruit development in these plants, which could make *SUP* genes a target for crop improvement.

## Dive Curated Terms

The following phenotypic, genotypic, and functional terms are of significance to the work described in this paper:

SUP Gramene: AT3G23130
SUP Araport: AT3G23130

plant cell AmiGo: PO:0009002

RUBY Gramene: AT1G19900
RUBY Araport: AT1G19900
